# Children with autism spectrum disorder and alterations in eating behavior: could it be gastroesophageal reflux disease?

**DOI:** 10.1016/j.jped.2025.101487

**Published:** 2025-12-17

**Authors:** Christine Audet de Almeida, Eduardo Sampaio Siqueira, Marcelo do Rego Maciel Souto Maior, Kátia Galeão Brandt

**Affiliations:** a Universidade Federal de Pernambuco (UFPE), Hospital das Clinicas, Recife, PE, Brazil; b Pós Graduação em Saúde da Criança e do Adolescente da Universidade Federal de Pernambuco, PE, Brazil; c Centro de Ciências Médicas da Universidade Federal de Pernambuco, PE, Brazil; d Pós Graduação em Nutrição da Universidade Federal de Pernambuco, PE, Brazil

**Keywords:** Eating behavior, Autism, Autism spectrum disorder, Child, Gastroesophageal reflux disease

## Abstract

**Objective:**

Describe the occurrence of warning signs of gastroesophageal reflux disease (GERD) and esophagitis in children with eating behavior (EB) alterations associated with autism spectrum disorder (ASD).

**Method:**

Descriptive study of 115 children aged 3 to 12 years, followed at a tertiary hospital and previously diagnosed with ASD. The BRCA-TEA instrument was applied to identify children with EB alterations, and the 17-ATN-GISSI instrument was applied to identify those with warning signs of GERD. The selected children were invited for a medical consultation to identify those with suspected esophagitis and an indication for upper gastrointestinal (GI) endoscopy with biopsies.

**Results:**

Sixty-nine children (60 %) were classified with alterations in the EB and, among these, 62 (89.8 %) presented warning signs of GERD. Eighteen children had suspected esophagitis and an indication for upper GI endoscopy. Among the 8 children who underwent the exam, 1 child had grade A erosive esophagitis, 1 child had grade B erosive esophagitis, and 1 child had eosinophilic esophagitis.

**Conclusion:**

A high frequency of EB alterations was found in children with ASD. The high frequency of GERD warning signs may be related to EB alterations in this group. Cases of esophagitis highlight the possibility of an organic disease. In these cases, performing upper GI endoscopy with biopsies is essential for diagnosis.

## Introduction

Eating is a necessary act for our survival, providing great pleasure, but it can also be disrupted by numerous factors. Eating habits in children are partially based on biological factors, but subject to environmental influences that can change or develop with age.^1^ Children with ASD have a high prevalence of alterations in EB, ranging from 49 % to 90 %.^2^ Alterations in EB in this group are usually associated with behavioral causes and are attributed to characteristics common to ASD, such as rigid and repetitive patterns of behavior, oral sensory difficulties, and impairment in social skills.^3^^,^^4^

Although behavioral causes are often given more importance in the literature, children with ASD also have a greater chance of developing gastrointestinal (GI) disorders and diseases, such as GERD and esophagitis, possibly explained by changes in the regulation of the brain-gut axis.^5^ There is also evidence that children with diseases of the upper gastrointestinal tract, such as GERD associated with esophagitis, are more likely to present alterations in the EB.^6^

The association between changes in eating behavior and esophagitis/GERD in neurotypical children is well established.^6^^,^^7^ This association appears to be less studied in children with ASD. Similarly, the occurrence of symptoms related to GERD is reported in the literature, but cases confirmed by complementary exams are rarer to be found.8., 9., 10. Using the keywords esophagitis/gastroesophageal reflux disease/autism/children, few articles were found related to histologically confirmed esophagitis.^11^^,^^12^

Professional societies' protocols recommend performing upper GI endoscopy to evaluate children with warning signs of GERD-associated esophagitis.^13^ However, the warning signs of GERD described in international protocols^13^ are useful for evaluating neurotypical children but have limited use in individuals with ASD and communication difficulties.^14^ Kara Gross Margolis, from Columbia University, and her collaborators developed, in 2019, an instrument to research warning signs of the most common gastrointestinal disorders in children with ASD,^15^ making screening of this group of patients more viable.

A better understanding of the factors involved in EB alterations in children with ASD, particularly the possibility of an organic disease such as esophagitis, motivated this study. It is understood that attention to this diagnosis can improve the management of these patients and improve the care provided to children with ASD and alterations in EB.

The overall objective of this study was to describe the occurrence of warning signs of GERD and esophagitis in children with ASD and alterations in EB. The specific aims were to identify the subgroup of patients with alterations in EB, among children with ASD; to characterize the occurrence of GERD warning signs in this subgroup, and to analyze the occurrence of esophagitis among children with GERD warning signs.

## Methods

This is a descriptive study conducted in outpatient clinics that treat children with ASD at the Hospital das Clínicas of the Universidade Federal de Pernambuco (UFPE) and at the Clínica Escola de Fonoaudiologia of UFPE. Data collection was from January to October 2024. Inclusion criteria were children aged 3 to 12 years, with a medical diagnosis of ASD previously established by a pediatric neurologist and/or child psychiatrist, and informed by the caregiver. Children with other disorders that could, in themselves, result in alterations in EB and/or GERD, such as cerebral palsy, malformation of the gastrointestinal tract, heart disease, or chronic lung disease, were excluded. The research was approved by the Human Research Ethics Committee of the Hospital das Clínicas - CEP of HC/EBSERH/UFPE under CAAE 74292023.9.0000.8807.

The children were classified as having or not having alterations in EB using the translated to Brazilian Portuguese version of the Brief Autism Mealtime Behaviour Inventory (BAMBI), named *Breve Registro de Comportamento Alimentar-Trantorno do Espectro do Autismo* (BRCA-TEA) instrument^16^ [Suppl. Material]. Among children with alterations in EB, those with warning signs of GERD were selected through the Autism Treatment Network Gastrointestinal Signs and Symptoms Inventory-17 (ATN-GISSI-17)^15^ [Suppl Material]. Finally, in another step, individuals with warning signs of GERD were evaluated during a medical consultation to identify those with clinical suspicion of esophagitis and indication for upper GI endoscopy, to confirm the diagnosis [Suppl Material].

The following were definitions used in the context of the present study:

Alterations in EB were assessed using the BRCA-TEA instrument and defined as present when the score obtained was greater than or equal to 47 out of a total of 90 points.^16^ The alterations in EB were categorized in three domains: food refusal, ASD characteristics, or limited variety.

GERD warning signs were assessed using the ATN-GISSI-17 instrument^15^ and defined as present when at least ONE of the warning signs for GERD described in items 2, 9, 10, 13 or 16 occurred, or TWO or more warning signs described in items 3, 11, 15 or 17.

Clinical suspicion of esophagitis was defined in a medical consultation, in accordance with international guidelines (Guidelines from ESPGHAN AND NASPGHAN for GERD and Eosinophilic Esophagitis) to analyze the clinical history and physical examination data^13^^,^^17^

Reflux esophagitis was defined by the presence of erosions or ruptures in the esophageal mucosa, or immediately above the esophageal gastric junction, observed on upper GI endoscopy, and characterized according to the Los Angeles classification.^13^^,^^18^

Eosinophilic esophagitis was defined by the presence of 15 or more eosinophils per high power field (HPF) observed in histology.^17^

The data were tabulated in Excel 2016 software, and the statistical analysis was performed by *Caves* version 2.4. To assess the association between categorical variables, the Exact test of *Fisher* and/or Chi-square *Pearson*. And for the comparison between two medians of independent samples, the U test *Mann-Whitney* was used. A significance level of 5 % was used to reject the null hypothesis.

## Results

**Figure 1:**
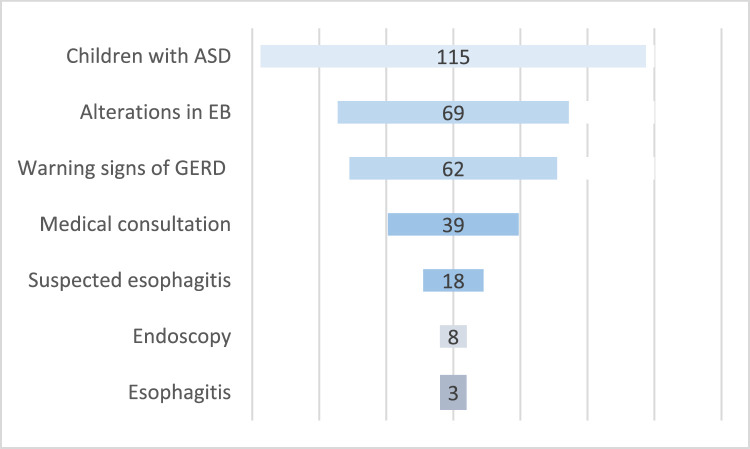
Number of children with ASD, alterations in EB, warning signs of GERD and esophagitis.

Of the 115 children who participated in the study, 69 (60 %) were classified as having alterations in EB. Among the individuals with alterations in EB, 62 (89.8 %) presented warning signs of GERD (Figure 1). Thirty-nine children (62.9 %) attended the medical appointment. Among these, 18 (46.1 %) had clinical suspicion of esophagitis and an indication for upper GI endoscopy. Thirteen children had the consent of their guardians to undergo upper GI endoscopy, but only 08 underwent the exam. In the end, 03 children (37.5 %) were diagnosed with esophagitis (01 erosive esophagitis grade A, 01 erosive esophagitis grade B, and 01 eosinophilic esophagitis) (Figure 1).

**Figure 2:**
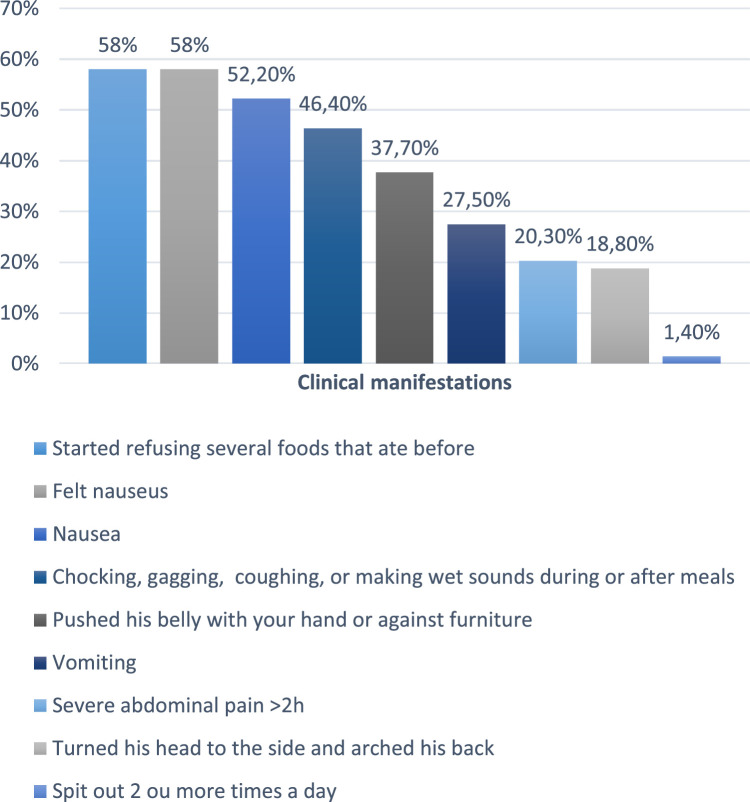
Warning signs of GERD in children with ASD and alterations in EB.

The sociodemographic characteristics of children with ASD in a tertiary hospital are described in Table 1. The warning signs of GERD are described in (Figure 2)**.** The clinical description, macroscopic and microscopic characteristics of the patients with esophagitis are described in Table 2.

**Table 1:** Sociodemographic characteristics of children with ASD in a tertiary hospital.

Variable	N total=115		With alterations in EB		Without alterations in EB		p
Age (years)			Med (IQ) ^d^ 5.0 (4.0 – 7.0)		Med (IQ) ^d^ 5.0 (4.0 – 8.0)		0.472^c^
	**N**	**%**	**N**	**%**	**N**	**%**	
**Sex**							
Male	90	78.3	55	79.7	35	76.1	0.652^b^
Female	25	21.7	14	20.3	11	23.9	
**Residence**							
Recife	57	49.5	36	52.2	21	47.7	0.898^b^
Metropolitan region	29	25.2	17	24.6	12	27.3	
Countryside	27	23.5	16	23.2	11	25.0	
**Interviewed’s educational level**							
Elementary school	22	19.1	13	18.8	9	19.6	0.791^b^
Hight school	76	66.1	47	68.1	29	63.0	
Higher education	17	14.8	9	13.0	8	17.4	
**Main caregiver**							
Mother	90	78.3	56	81.2	34	73.9	0.356^a^
Others	25	21.7	13	18.8	12	26.1	
**Diagnosis of ASD**							
Child neurologist	89	77.4	51	73.9	38	82.6	0.459^a^
Child psychiatrist	22	19.1	16	23.2	6	13.0	
Both	4	3.5	2	2.9	2	4.3	

(a) Fisher's exact, (b) Chi-square test and (C) Mann-Whitney U test (d) Median and interquartile range.

**Table 2:** Clinical description, macroscopic and microscopic characteristics of children with ASD , EB and esophagitis.

PACI ENT	AGE	S	ALTERATIONS IN EB	GERD	ESOPHA GITIS	MACROSCOPIC ALTERATIONS	MICROSCOPIC ALTERATIONS
J.V.P.L	6 years	M	Turns face away or closes mouth when food is offered, spits out food, has unruly behavior during meals, food selectivity, refuses foods that require a lot of chewing, prefers crunchy foods	Vomiting, gagging, choking, coughing during meals	Eosinophilic esophagitis	Exudate, white patches, or plaques on >10 % of the esophageal surface area and vertical grooves with visible depth. Conclusion: Suggestive of eosinophilic esophagitis	Findings: 60 eosinophils/HPF, hyperplasia of the epithelial basal layer, abscess or eosinophilic aggregate in the epithelium, superficial alignment and eosinophils in the upper 1/3 of the epithelium, dilation of the intercellular spaces of the epithelial cells, epithelial dyskeratosis and fibrosis of the lamina propria. Conclusion: Eosinophilic esophagitis
A.H.A.A	3 years	M	Cries or screams during meals, refuses foods that require a lot of chewing, food selectivity, has unruly behavior during meals, prefers crunchy foods	Nausea, vomiting, Sandifer Syndrome, choking	Reflux esophagitis	Isolated esophageal erosion greater than 5mm Conclusion: Los Angeles Grade B Erosive Esophagitis	Basal layer hyperplasia, papillary elongation, vascular growth, dilation of the intercellular spaces of epithelial cells Conclusion: Suggestive of reflux esophagitis
M.C.A.C	10 years	M	Cries or screams during meals, turns away or spits out food, has unruly behavior during meals, food selectivity, refuses foods that require a lot of chewing, is aggressive during meals, has no interest in food, eats little, prefers crunchy foods, prefers sweet foods	Nausea, Sandifer Syndrome, pushing the belly with hand	Reflux esophagitis	Esophageal erosion of no >5mm Conclusion: Los Angeles Grade A Erosive Esophagitis	Basal layer hyperplasia, papillary elongation Conclusion: Suggestive of reflux esophagitis

Among the five children who underwent upper GI endoscopy but demonstrated no abnormalities on either macroscopic or microscopic evaluation, several exhibited warning signs consistent with gastroesophageal reflux disease (GERD). These clinical manifestations were indistinguishable from those observed in patients with abnormal findings. Reported symptoms included nausea in five children, abdominal pain in four, refusal to consume previously accepted foods in four, choking, gagging, coughing, or producing wet vocalizations during or after meals in four, head-turning and back-arching in one, and abdominal pressure applied by hand or against furniture in one child.

## Discussion

The current study observed that 69 (60 %) of 115 children with ASD presented alterations in EB. Among the individuals with alterations in EB, 62 (89.9 %) also presented warning signs of GERD. In the end, among the 08 children who underwent upper GI endoscopy, 03 (37.5 %) were diagnosed with macroscopic and microscopic esophagitis.

The high frequency of alterations in EB observed in children with ASD in this study is consistent with rates presented in other publications, with frequencies ranging from 49 % to 90 % .^2^

Upper gastrointestinal disorders, especially GERD-related esophagitis, may be associated with alterations in EB in children.^6^ However, these disorders are underappreciated, both in the literature and in clinical practice, when evaluating children with ASD and alterations in EB. Often, the focus of attention in this group is on the behavioral causes of alterations in EB.^3^^,^^4^

The current study found a very high frequency of GERD warning signs among children with ASD and alterations in EB. Screening instruments for warning signs of GERD in typical or atypical children generally have low specificity. However, the real possibility of a higher frequency of GERD in the group of children with ASD cannot be ignored, as some other studies also suggest this possibility. A study of children with ASD in Italy, without determining the occurrence of alterations in EB, showed that approximately 40 % of the sample had GERD warning signs, making GERD the second most common gastrointestinal disorder, behind only constipation.^19^

There is biological plausibility for the higher incidence of GERD in individuals with ASD. The high prevalence of gastrointestinal disorders in ASD has led researchers to investigate a genetic association between brain and gut disorders in this group. The enteric nervous system (ENS) is described as our "second brain" due to its many similarities with the central nervous system (CNS). Furthermore, the ENS and CNS can communicate continuously and bidirectionally. Studies in animal models demonstrate the possibility of genes involved in autistic traits and various alterations in gastrointestinal function.^5^^,^20., 21., 22.

GERD occurs when, in the presence of gastroesophageal reflux, there is an imbalance between the aggressive and defensive mechanisms of the esophageal mucosa. The refluxed material in the esophagus causes inflammation of the mucosa, known as esophagitis. This inflammatory process can sensitize nociceptors, causing symptoms of hypersensitivity and dysmotility.^23^ Esophagitis associated with GERD is the most common organic cause of pain during feeding. If a child experiences pain, nausea, fatigue, gagging, coughing, or choking while feeding, they may learn to associate this moment with unpleasant experiences.^6^^,^^7^

In the current study, 37.5 % of children with ASD and EB alterations who underwent upper GI endoscopy had esophagitis, including two cases of erosive esophagitis and one case of eosinophilic esophagitis. Research investigating the prevalence of erosive esophagitis in children, unlike in adults, is still limited, and studies involving individuals with ASD are few. A retrospective study conducted in Boston compared the clinical and endoscopic characteristics of 2,014 pediatric patients with and without ASD. The results showed that children with ASD had a higher frequency of esophagitis (38.4 %), compared to children with developmental delay unrelated to ASD (33.4 %) and children with typical development (30.4 %) (*P* = 0.008).^11^

In the current study, one child was identified with eosinophilic esophagitis. Although eosinophilic esophagitis is a rarer esophageal disease than GERD, it has also been identified more frequently in children with ASD compared to neurotypical children. A study conducted in the United States with 45,286 children with ASD and 226,430 control children showed a higher occurrence of eosinophilic esophagitis in children with ASD (0.4 % vs. 0.1 %). Alterations in EB were strongly associated with eosinophilic esophagitis in both groups.^12^

Screening tools can be very useful in identifying children who require further investigation. Understanding the symptoms expressed in the "language" of children with ASD may help to identify, possibly more frequently, GERD in this group. In the current study, the tool adapted for children with ASD^15^ was able to identify individuals with warning signs of GERD who were subsequently referred for medical consultation.

The study has some limitations. It was necessary to use articles containing different nomenclatures for childhood feeding problems (alterations in EB, feeding problem, feeding difficulty, or eating disorder) as bibliographic references. This was due to the lack of consensus in the current scientific literature, where the various terms are used interchangeably. Only some of the children with warning signs of GERD attended a medical appointment, limiting the identification of other individuals who might require upper GI endoscopy. It was also not possible to perform upper GI endoscopy on all children with a medical indication.

The study found a high frequency of alterations in EB in children with ASD, and among these, a high frequency of warning signs of GERD was also found. Food refusal characteristics were also significant in the group with EB alterations. Esophagitis, a condition that leads to pain and discomfort during eating and possible food refusal, can be considered one of the causes of the problem. The cases of esophagitis found highlight problems that had not been previously considered in this group of children with ASD and alterations in EB.

The authors recommend the development of a standardized protocol for the assessment of GERD and esophagitis in children with autism spectrum disorder. This protocol should clearly define the clinical indications for performing upper GI endoscopy. Based on current findings, it is anticipated that endoscopy may be warranted more frequently than is presently practiced.

## Funding

There was no funding source.

## Data availability statement

Data that support the findings of this study are available from the corresponding author.

## Conflicts of interest

The authors declare no conflicts of interest.

## Appendix Supplementary materials

Image, application 1

## Footnotes

Supplementary material associated with this article can be found in the online version at doi:10.1016/j.jped.2025.101487.
