# Supplementary material for: Children with autism spectrum disorder and alterations in eating behavior: could it be gastroesophageal reflux disease?
Source: J Pediatr (Rio J). 2025 Dec 17;102(1):101487. doi: 10.1016/j.jped.2025.101487 (PMC12774737; doi:10.1016/j.jped.2025.101487)
Supplement: Supplementary file 1 [file mmc1.docx]

**JPED-D-25-00299**

**Supplementary Material**

**BRCA-ASD SCALE FOR ASSESSING EATING BEHAVIOR IN ASD**

Often

Almost always/always

Rarely

Never

Occasionally

Often

The child....

| 1. cries or screams during meals | 1 | 2 | 3 | 4 | 5 |  |
| --- | --- | --- | --- | --- | --- | --- |
| 2. turns his face or body away when I offer him food | 1 | 2 | 3 | 4 | 5 |  |
| 4. spits the food out of the mouth | 1 | 2 | 3 | 4 | 5 |  |
| 7. has unruly behavior during meals | 1 | 2 | 3 | 4 | 5 |  |
| 8. close your mouth tightly when food is offered  **Total score for food refusal** | 1 | 2 | 3 | 4 | 5 |  |
|  |  |  |  |  |  |  |
| 3. remain seated at the table until the end of the meal 1 | | 2 | 3 | 4 | 5 |  |
| 5. is aggressive during meals 1 | | 2 | 3 | 4 | 5 |  |
| 6. exhibits self-harming behavior during meals 1 | | 2 | 3 | 4 | 5 |  |
| 9. is flexible with eating routines 1 | | 2 | 3 | 4 | 5 |  |
| 12. refuses to eat foods that require a lot of chewing 1  **Total score for ASD characteristics** | | 2 | 3 | 4 | 5 |  |

| 10. is willing to try new foods | 1 | 2 | 3 | 4 | 5 |  |
| --- | --- | --- | --- | --- | --- | --- |
| 11. does not like certain foods and does not consume them | 1 | 2 | 3 | 4 | 5 |  |
| 13. prefer the same foods at every meal | 1 | 2 | 3 | 4 | 5 |  |
| 14. prefers crunchy foods | 1 | 2 | 3 | 4 | 5 |  |
| 15. accepts or prefers a variety of foods | 1 | 2 | 3 | 4 | 5 |  |
| 16. prefer food served in a specific way | 1 | 2 | 3 | 4 | 5 |  |
| 17. prefers sweet foods | 1 | 2 | 3 | 4 | 5 |  |
| 18. prefers foods prepared in a specific way  **Total score for limited variety** | 1 | 2 | 3 | 4 | 5 |  |

Não tenho certeza

**TOTAL SCORE (greater than or equal to 47 = change in EB**

Castro *et al*. (2019)

**Autism Treatment Network GI Signs & Symptoms Inventory-17 (ATN-GISSI-17)**

Child’s Name _____________________________________Today’s Date: ___ / ___ / 2 0

Child’s Gender ____ Age ___ years Child’s Date of Birth / ___ / __

Your relationship to this child: Mother Father Other:

**Most of the questions on this form are about THE LAST THREE MONTHS. Please put a check (
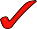
) in the box that best describes your child.**

| Yes | No | Unsure |
| --- | --- | --- |

**In the last 3 months**, has your child experienced any of the following gastrointestinal (tummy) symptoms:

1. Abdominal (belly) pain
2. Nausea
3. **In the last year,** did your child have severe gastrointestinal (tummy) pain that lasted 2 hours (or longer) and caused your child to stop all activities?
4. **In the last 3 months,** how often did your child usually have BMs?
   1. Less than once a week
   2. 1-2 Times a week
   3. 3-6 Times a week
   4. Once a day
   5. 2-3 Times a day
   6. More than 3 times a day
   7. Unsure
5. **In the last 3 months**, what were your child’s BMs usually like?
   1. Very hard
   2. Hard
   3. Not too hard and not too soft
   4. Very soft or mushy
   5. Watery
   6. Unsure

| Yes | No | Unsure |
| --- | --- | --- |

**In the last 3 months, did your child:**

1. Appear to feel pain when having a BM?
2. Have to rush to the bathroom for a BM?
3. **Has your child ever** had a black, tarry BM?

## **In the last 3 months, has your child:**

1. Spit up ≥ 2x per day?
2. Experienced retching?
3. Tilted his/her head to the side and arched back?

## **In the last 3 months, has your child missed activities due to:**

1. pain and/or discomfort?
2. vomiting
3. problems with BMs
4. **In the last 3 months,** did your child push his abdomen with his/her hands or your hands, push his/her abdomen against or lean forward over furniture?
5. **In the last 3 months,** did your child choke, gag, cough, or sound wet during or after swallowing or with meals?
6. **In the last 3 months,** has your child started to refuse many foods that he or she would eat in the past?

(Margolis et al, 2019)

ATN-GISSI-17 Score Sheet

Directions: For any “yes” or checked response above, place a 🗸 next to the corresponding item number in the columns below.

| **Functional Constipation** | **Functional Diarrhea** | **GERD** | **GERD** |
| --- | --- | --- | --- |
| 1. | 2. | 2. | 3. |
| 4a. | 5d. | 9. | 11. |
| 4b. | 5e. | 10. | 15. |
| 5a. | 7. | 13. | 17. |
| 5b. | 8. | 16. |  |
| 6. | 11. |  |  |
| 12. | 14. |  |  |
| 14. | 17. |  |  |
| 17. |  |  |  |
| 🗸 in column above? | 🗸in the column above? | 🗸 in the column above? | 2 or more 🗸 |
| if yes, then screen positive | if yes, then screen positive | if yes, then screen positive | in the column |
| for **functional constipation** | for **functional diarrhea** | for **GERD** | above? Then |
|  |  |  | also screen |
|  |  |  | positive for |
|  |  |  | **GERD** |

| **FORM FOR MEDICAL CONSULTATION OF CHILDREN AND ADOLESCENTS WITH WARNING SIGNS OF GERD** | | | |
| --- | --- | --- | --- |
| **NAME:** | | | |
| **DATE OF BIRTH:** | | **AGE:** | |
| **CAREGIVER:** | | | |
| **MAIN COMPLAINT AND HISTORY OF PRESENT ILLNESS** | | | |
| **According to the Pediatric Gastrointestinal Endoscopy: ESPGHAN and ESG guidelines and the indications for Upper GI endoscopy in suspected esophagitis, did your child present any of the following signs or symptoms below?**  Weight loss  Yes ( ) No ( ) I don’t know ( )  Unexplained anemia  Yes ( ) No ( ) I don’t know ( )  Abdominal pain with suspected organic disease  (Abdominal pain further from the navel and better located, in the epigastric region, which may wake the child or adolescent at night or interrupt pleasurable activities, or which may be associated with eating.  Yes ( ) No ( ) I I don’t know ( )  Dysphagia (difficulty swallowing)?  Yes ( ) No ( ) I don’t know ( )  Odynophagia (pain when swallowing)?  Yes ( ) No ( ) I don’t know ( )  Chest pain ?  Yes ( ) No ( ) I don’t know ( )  Recurrent vomiting related to food?  Yes ( ) No ( ) I don’t know ( )  Hematemesis (vomiting with blood)?  Yes ( ) No ( ) I don’t know ( )  Hematochezia or melena (bright or dark red blood in the stool)?  Yes ( ) No ( ) I don’t know ( )  Difficulty with eating?  Yes ( ) No ( ) I don’t know ( )  **Escore de sintomas de ESOFAGITE EOSINOFÍLICA PEDIÁTRICA (relatório para pais de crianças e adolescentes de 2 a According to the Pediatric Gastrointestinal Endoscopy: ESPGHAN and ESG guidelines and the indications for Upper GI endoscopy in suspected esophagitis, did your child present any of the following signs or symptoms below?**  **weight loss**  **Yes ( ) No ( ) I don’t know ( )**  **Unexplained anemia**  **Yes ( ) No ( ) I don’t know ( )**  **Abdominal pain with suspected organic disease**  **(Abdominal pain further from the navel and better located, in the epigastric region, which may wake the child or adolescent at night or interrupt pleasurable activities, or which may be associated with eating.**  **Yes ( ) No ( ) I don’t know ( )**  **Dysphagia (difficulty swallowing)?**  **Yes ( ) No ( ) I don’t know ( )**  **Odynophagia (pain when swallowing)?**  **Yes ( ) No ( ) I don’t know ( )**  **Chest pain?**  **Yes ( ) No ( ) I don’t know ( )**  **Recurrent vomiting related to food?**  **Yes ( ) No ( ) I don’t know ( )**  **Hematemesis (vomiting with blood)?**  **Yes ( ) No ( ) I don't know ( )**  **Hematochezia or melena (bright or dark red blood in the stool)?**  **Yes ( ) No ( ) I don't know ( )**  **Difficulty with eating?**  **Yes ( ) No ( ) I don't know ( )**  **PEDIATRIC EOSINOPHILIC ESOPHAGITIS Symptom Score (report for parents of children and adolescents aged 2 to 18 years) adapted from the Pediatric Eosinophilic Esophagitis Symptom Score (PEESS v2.0) translated and adapted for Brazilian culture (Santos et al., 2018).**  **How often does your child:**  **• Have chest pain?**  **• Have heartburn (burning in the chest, mouth, or throat)?**  **• Have stomach or belly pain?**  **• Have difficulty swallowing?**  **• Feel like food is stuck in their throat or chest?**  **• Need to drink something to help swallow the food?**  **• Vomit?**  **• Feel nauseous (feel like you're going to vomit, but don't vomit?)**  **• Feel like food comes back up in your throat when you eat?**  **• Eat less than other people?**  **• Need more time to eat than other people?**  **Frequency score (0, 1, 2, 3, 4): never, rarely (1 to 2 times/month), sometimes (1 to 2 times/week), often (1x/day), and almost always (2 or more times/day)**  **Severity score 0, 1, 2, 3, 4 (how they feel in these situations):**  **Not bad, a little bad, moderately bad, bad, or very bad**  **Has your child ever had food impaction?** | | | |
| **DETAIL THE INFORMATION:** | | | |
| **IS (SYMPTOMATOLOGICAL INTERROGATION):**  **General:**  **ACV:**  **AR:**  **AD:**  **AGU:**  **AME:**  **Skin:**  **SN:**  **PERSONAL HISTORY:** | | | |
| **PERSONAL BACKGROUND:** | | | |
| **FAMILY BACKGROUND::** | | | |
| **FOOD:**  **USE OF MEDICATIONS? Yes ( ) No ( )**  **Wich ?** | | | |
| **MULTIDISCIPLINARY THERAPIES:** | | | |
| **WEIGHT:** | **HEIGHT:** | | **BMI:** |
| **PHYSICAL EXAMINATION:** | | | |
| **DIAGNOSTIC HYPOTHESIS:** | | | |
| **UPPER GI ENDOSCOPY INDICATION? Yes ( ) No ( )** | | | |
| **ORIENTATION:** | | | |
